# Roasting Improves the Bioaccessibility and Bioactivity of Polyphenols from Highland Barley with a Protective Effect in Oxidatively Damaged HepG2 Cells

**DOI:** 10.3390/foods14122095

**Published:** 2025-06-14

**Authors:** Nuo Chen, Shuyu Pang, Xingru Zao, Qin Luo, Lingyuan Luo, Wenming Dong, Yongqiang Li

**Affiliations:** 1 College of Food Science and Technology, Yunnan Agricultural University, Kunming 650201, China; 13367608945@163.com (N.C.); shuyupang0715@163.com (S.P.); 15126607035@163.com (L.L.); 2 Miyi County Agriculture and Rural Bureau, Panzhihua 617200, China; 3 College of Agricultural and Food Engineering, Baise University, Baise 533000, China; luoqin1526@163.com

**Keywords:** highland barley, polyphenols, roasting, bioaccessibility, bioactivity, oxidative damage

## Abstract

This research is designed to explore the effect of roasting on the release, bioaccessibility, and bioactivity of polyphenols in highland barley (HB). The findings of in vitro digestion indicated that roasting significantly improved the bioaccessibility of polyphenols in HB flour (gastrointestinal digestion stage: raw HB: 187.28%, roasted HB: 285.65%; colonic fermentation stage: raw HB: 188.13%, roasted HB: 255.36%) and enhanced its antioxidant activity. Moreover, the inhibitory impacts of polyphenols on the activities of α -amylase, α-glucosidase, and lipase mainly occur in the small intestine. Roasting increased inhibitory activities of polyphenols on α-amylase, α-glucosidase, and lipase in the small intestine (*p* < 0.05), with IC50 values of 71.31 ± 1.35 μg FAE/mL, 60.44 ± 1.35 μg FAE/mL, and 52.94 ± 2.51 μg FAE/mL, respectively. HepG2 cells, a human hepatocellular carcinoma cell line, are commonly employed in oxidative stress and antioxidant studies due to their ability to mirror the protective effects of bioactive compounds against oxidative damage in liver cells. This study aimed to establish a model of H_2_O_2_-induced oxidative stress injury in HepG2 cells and to evaluate the protective effect of digested HB polyphenol extract against oxidative injury. It was found that the polyphenols extracted from roasted HB help reduce reactive oxygen species (ROS) and malondialdehyde (MDA) through increased activities of superoxide dismutase (SOD), glutathione (GSH), catalase (CAT), glutathione peroxidase (GPx), and total antioxidant capacity (T-AOC), thereby providing enhanced defense against oxidative damage in HepG2 cells. The findings of this research pave the way for the development of new functional foods utilizing roasted HB.

## 1. Introduction

Highland barley (HB), a common barley species belonging to the *Poaceae* family, is primarily cultivated in the Qinghai–Tibet Plateau [1]. Furthermore, HB is the only crop that can be successfully grown in a rough environment characterized by low temperature and oxygen, high radiation, and intense ultraviolet light, which promotes the formation of an excellent reservoir of polyphenols, fiber, and β-glucan, all of which are endowed with various health-promoting effects [2,3].

Tsampa is a traditional staple food for Tibetan residents, which has been proven to possess a variety of health benefits [4]. Typically, tsampa is made from roasted HB flour, salted butter tea, and milk residue, among which roasting of HB may be a key step in promoting the release of bioactive and aroma compounds, as well as facilitating the ready-to-eat product [5]. Current information about roasting for improving the release of polyphenols from HB and their bioaccessibility is scarce.

Polyphenols can be classified into two categories according to their solubility in the extraction medium: soluble polyphenols (SPs) and insoluble bound polyphenols (IBPs) [6]. It has been proposed that polyphenols provide multiple health benefits, like antioxidant activity, anti-obesity, and alleviation of oxidative stress [7,8]. SPs can be easily extracted and collected by aqueous-organic solvents. By contrast, IBPs may be difficult to extract and obtain using the extraction method mentioned above, as they are tightly linked to cellulose, hemicellulose, pectin, and structural protein by covalent bonds and non-covalent interactions [9]. It has been indicated that polyphenols need to be released from the food materials through digestion to become bioaccessible and exert their beneficial effects [10]. Some researchers have put forward the idea that thermal treatments could improve the extractability of IBPs by breaking down the intricate bonds between polyphenols and dietary fiber or protein, as well as loosening the grain matrix, which results in an increase in total polyphenol content (TPC) and their antioxidant potential [3,11]. Additionally, many studies reported that thermal treatment can promote the release of IBPs and improve the bioaccessibility and bioactivity of polyphenols in black beans [12], oat bran [13], and millet [14] during in vitro digestion.

Oxidative stress, characterized by an imbalance between the generation of free radicals and the antioxidant defenses, is a key driver of cellular damage and inflammation, making a considerable impact on the progression of long-term illnesses like heart disease, diabetes, and cancer [15]. HepG2 cells, a human hepatoma cell line, are extensively utilized in oxidative stress and antioxidant research due to their metabolic properties closely resembling those of normal hepatocytes [16]. Numerous in vitro studies have identified polyphenols as powerful exogenous antioxidants that can neutralize oxidative stress mediators while also upregulating the activities of endogenous antioxidant enzymes, thereby effectively alleviating intracellular oxidative damage [15,17].

It is commonly believed that the health-promoting effects of polyphenols are heavily reliant on their bioavailability, which is closely related to their bioaccessibility in the digestive tract [18,19]. Currently, in vitro digestion models and in vivo experiments have been extensively utilized to assess the bioaccessibility of polyphenols in food substrates. Compared with in vivo experiments, in vitro digestion models, including in vitro gastrointestinal digestion (GID) and colonic fermentation (CF), provide numerous advantages, including rapidity, efficiency, cost-effectiveness, low labor intensity, and the absence of ethical constraints [20]. Previous research has confirmed that in vitro digestion models can successfully elucidate the release of polyphenols from carob [18], carrots [20], and corn [21], as well as the subsequent changes in bioaccessibility and bioactivity of polyphenols.

Previously, some laboratory studies investigated the bioaccessibility of polyphenols in roasted HB by following an in vitro gastrointestinal digestion process [3]. However, limited research has focused on the role of roasting in the release of polyphenols from HB and the bioaccessibility and bioactivity of these compounds throughout the process involving in vitro gastrointestinal digestion and colonic fermentation. In our research, we explored the role of roasting in promoting the release of polyphenols from HB and further assessed their bioaccessibility and bioactivity. Furthermore, we evaluated the protective effects of digested SPs against H_2_O_2_-induced oxidative damage in HepG_2_ cells.

## 2. Materials and Methods

### 2.1. Chemicals and Materials

Sodium carbonate anhydrous, Folin–Ciocalteu reagent, hydrochloric acid, sodium hydroxide, ethyl acetate, diethyl ether, soluble starch, Trolox, and hydrogen peroxide (H_2_O_2_). Phenolic standards were procured from Shanghai Yuanye Biotechnology Co., Ltd. (Shanghai, China). Enzymes such as porcine pancreatic α-amylase, α-glucosidase, and lipase were sourced from Sigma-Aldrich (St. Louis, MO, USA). Additionally, the human hepatocellular carcinoma cell line HepG2 was obtained from the Cell Bank of the Chinese Academy of Sciences (Shanghai, China).

### 2.2. Preparation of Roasted HB

HB was purchased from the Agricultural Science Institute of Diqing Tibetan Autonomous Prefecture (Yunnan, China) and stored at 4 °C. A hundred grams of HB was soaked in water for 24 h according to the traditional methods for producing tsampa, with the water level submerged below the HB. Subsequently, the moisture content of HB was reduced to between 15% and 17% through air-drying at room temperature for 24 h (the air-drying time of the sample was determined based on the preliminary experiment), which improved the quality and texture of the final product. The moisture content was measured using a moisture analyzer (Kern DBS, Balingen, Germany). Afterward, HB was roasted at 220 °C for about 10 min by an induction cooker (RT2134, Midea Group, Shanghai, China) until the percentage of popped grain reached 85%. The roasted HB was milled with a pulverizer and then passed through a 60-mesh sieve. After that, a portion of the HB flour was directly subjected to an in vitro digestion. Another portion of the HB flour was freeze-dried and subsequently stored at −80 °C until the morphological property analysis. Raw HB flour served as a control.

### 2.3. Morphological Property Analysis of HB

#### 2.3.1. Scanning Electron Microscope (SEM)

The freeze-dried HB flour was subjected to grinding and then passed through a 100-mesh sieve. The surface microstructural features of the flour were analyzed using scanning electron microscopy (SEM, Thermo Fisher Scientific, Waltham, MA, USA). For detailed observation of the surface microstructures, images were captured at magnifications of 500× and 1000× using an FEI-NOVA Nano SEM 230 instrument(FEI, Hillsboro, OR, USA).

#### 2.3.2. X-Ray Diffraction (XRD)

The structural analysis of HB samples was performed utilizing a Bruker D8 Advance X-ray powder diffractometer from Japan, which was operated at 40 kV and 40 mA. Scanning of the samples occurred across a 2θ interval from 5° to 90°, with a scan rate set at 5° per minute. MDI Jade 6.0 software (MDI, Livermore, CA, USA) was employed to assess the relative crystallinity.

#### 2.3.3. Fourier Transforms Infrared (FT-IR) Spectroscopy

FT-IR spectroscopy was conducted utilizing an FT-IR spectrometer (Thermo Fisher Scientific, Waltham, MA, USA). The preparation of the sample involved blending it with potassium bromide (KBr) and then forming thin pellets through compression. Spectral analysis was executed over a wavenumber range spanning 400 to 4000 cm^−1^, with each spectrum derived from the accumulation of 32 scans at a resolution of 4 cm^−1^.

### 2.4. Simulated In Vitro Digestion

#### 2.4.1. In Vitro Gastrointestinal Digestion

This experiment was modified from a previous procedure [22] and included three phases: oral, gastric, and intestinal. For the oral phase, 5.0 g of sample was combined with 5.0 mL of saliva (collected from 6 healthy individuals) at a 1:1 (*w*/*v*) ratio; then, nitrogen was injected, and the mixture was homogenized and incubated at 37 °C for 2 min. The samples were gathered and freeze-dried at −80 °C for further analysis. For the gastric phase, a solution containing simulated 8 mL gastric fluid (SGF), 6000 U/mL 0.667 mL pepsin, 5 μL CaCl_2_, 5 M 0.4 mL HCl, and 0.928 mL distilled water was combined with mixtures in the oral step at a 1:1 (*v*/*w*) ratio; then, nitrogen was injected, and the mixture was incubated at 37 °C for 2 h. The samples were gathered and freeze-dried at −80 °C for further analysis. To complete intestinal digestion, 0.8 mL of NaOH (5 M) was subsequently introduced to the above digestive phase to adjust the pH to 7.0. The intestinal phase comprised 8 mL simulated intestinal fluid, 133.4 mM 3 mL bile salts, 0.3 M 40 μL CaCl_2_, and 800 U/mL 5 mL pancreatin. The ratio of intestinal mixture to gastric phase is 1:1 (*v*/*w*). After nitrogen injection, the sample was shaken and incubated in the dark for 2 h (37 °C, 120 rpm). The samples were collected and freeze-dried at −80 °C for further analysis.

#### 2.4.2. In Vitro Colonic Fermentation

After the gastrointestinal simulation, the residue was processed through in vitro colonic fermentation using methods adapted from a previous method [23]. The fresh human fecal samples were donated by 6 healthy participants, including three males and three females, aged 20 to 25, with a BMI between 18.5 and 23. The volunteers were asked not to take antibiotics for 3 months and to avoid foods containing a high content of polyphenols and antibiotics for 48 h before sample collection. Next, to prepare a 32% (*w*/*v*) fecal suspension, the fresh fecal sample was subsequently mixed and homogenized in 0.1 M sterile phosphate-buffered saline (PBS) at a pH of 7.0, with additional inoculation of in vitro colonic fermentation medium and residual sample obtained following gastrointestinal digestion and subsequent centrifugation as described by the previous method [23]. Samples were obtained at different time points (0.5, 4, 8, 12, 16, 20, and 24 h) and then promptly stored at −80 °C for further analysis.

Bioaccessibility (%)=PCA/PCB×100

where *PCA* reflects the levels of polyphenols, flavonoids, or antioxidant capacity in the supernatant after in vitro digestion, while *PCB* reflects the corresponding levels of polyphenols, flavonoids, or antioxidant capacity before digestion.

### 2.5. Extraction of Polyphenols

SPs and IBPs were extracted from HB using previously described methods [24], with slight modifications. Summarily, 2.0 g of HB was extracted with 20 mL of methanol/formic acid/water (80:0.1:19.9, *v*/*v*/*v*) using an ultrasonic extractor at 4 °C for 1 h and then centrifuged at 8000 rpm at 4 °C for 15 min. This procedure was repeated once, and the resulting supernatant indicated the soluble fraction.

To obtain IBPs, the residue of SPs was treated by adding a 4 M NaOH solution for 4 h at room temperature under a nitrogen atmosphere, subsequently adjusting the pH to 2.0 using a 6 M HCl solution. Following this, the solution underwent centrifugation at 4 °C for 15 min at 8000 rpm. After centrifugation, the supernatant was transferred into a separatory funnel, and an equal volume of a 1:1 (*v*/*v*) mixture of diethyl ether and ethyl acetate was incorporated. The funnel was sealed and gently shaken to ensure thorough mixing and phase contact, followed by settling to allow for phase separation. The aqueous layer was carefully drained, and the organic layer was gathered. This process was repeated three times, and the combined organic layers were set aside for further processing. Finally, the solutions obtained were concentrated using rotary evaporation. All samples were kept at 4 °C until they were used.

### 2.6. Total Polyphenol Content (TPC) and Total Flavonoid Content (TFC) Measurement

#### 2.6.1. Measurement of TPC

This experiment was assessed following the previous procedure [24], with a slight modification. Briefly, a volume of 1.0 mL of polyphenol extract was combined with 0.25 mL of Folin–Ciocalteu reagent, followed by the addition of 3.25 mL of distilled water and 0.5 mL of a saturated sodium carbonate (Na_2_CO_3_) solution. After a 35 min incubation in darkness, the absorbance at 725 nm was recorded.

#### 2.6.2. Measurement of TFC

The TFC was assessed following the procedure reported by the previous method [24]. In short, the NaNO_2_ solution (0.3 mL, 5%) was introduced to the polyphenol extract (3.4 mL) and distilled water (3.4 mL). The mixture was kept in the dark to react at room temperature for 5 min. Afterward, a 10% AlCl_3_ solution was introduced, and the solution was further incubated in the dark for 1 min at room temperature. The solution was thoroughly mixed with 2 mL of 1 M NaOH, then incubated in the dark at room temperature for 15 min, after which the absorbance was measured at 510 nm.

### 2.7. Quantitative Analysis of Polyphenols

The high-performance liquid chromatography (HPLC) conditions for polyphenols were performed following our previous methods [2]. An amount of 20 μL of polyphenol extract was injected into the HPLC system (Poros R2 120 EC-C18 column: 150 mm × 4.6 mm, particle size: 4 μm). The mobile phase consisted of methanol (A) and 0.1% formic acid in water (B), a flow of 0.8 mL/min, a column temperature of 30 °C, and an injection volume of 20 μL. Detection was performed at 280 nm as the appropriate wavelength, utilizing a variable wavelength detector (VWD). The linear gradient elution was as follows: 0–7 min, 15% A to 85% B; 7–13 min, 20% A and 80% B; 13–20 min, 20% A and 80% B; 20–30 min, 30% A to 70% B; 30–35 min, 65% A to 35% B.

### 2.8. Assessment of Antioxidant Capacity

#### 2.8.1. DPPH Radical Scavenging Capacity

The assessment was performed based on the method described in [25]. In essence, 0.5 mL of polyphenols was combined with 2.0 mL of a DPPH solution (0.079 mM in methanol) and then incubated at room temperature in the dark for 30 min. Following this incubation, the absorbance was assessed at a wavelength of 517 nm. The results were determined using the equation provided below:

DPPH radical scavenging activity (%) = [(*A_c_* − *A_c’_*) − (*A_s_* − *A_s’_*)]/(*A_c_* − *A_c’_*) × 100

where *A_c_* is the absorbance of DPPH solution; *A_c’_* is the absorbance of methyl alcohol; *A_s_* is the absorbance after the reaction of DPPH with the polyphenol extract, and *A_s’_* is the absorbance after mixing methyl alcohol with the polyphenol extract.

#### 2.8.2. Ferric-Reducing Antioxidant Power (FRAP)

The assay was performed based on the procedures described in [26]. In brief, a ferric–TPTZ solution was formulated by combining sodium acetate buffer (300 mM, pH 3.6), FeCl_3_•6H_2_O (20 mM), and TPTZ solution (10 mM in 40 mM HCl) in a volume ratio of 10:1:1 (*v*/*v*/*v*). This solution was preheated to 37 °C for 20 min prior to use. Subsequently, 100 µL of polyphenols was combined with 3.0 mL of the preheated ferric–TPTZ solution, and the absorbance was recorded at 593 nm.

#### 2.8.3. ABTS Radical (ABTS•+) Scavenging Capacity

This experiment was conducted following the methodology by [24], with minor alterations. In summary, a solution of ABTS (7 mM) was combined with a potassium persulfate solution (2.45 mM) in a 1:1 volume ratio and stored in a brown bottle in the dark for a period of 12 to 16 h to create a reserve solution of ABTS•+. Afterward, the adjusted ABTS•+ working solution (3.8 mL) was added to 100 µL of polyphenols, and then, the combination was incubated for 6 min at room temperature. The absorbance was recorded at 734 nm. The results were computed using the following equation:

ABTS radical scavenging activity (%) = [(*A_c_* − *A_c’_*) − (*A_s_* − *A_s’_*)]/(*A_c_* − *A_c’_*) × 100

where *A_c_* is the absorbance of ABTS•+ working solution; *A_c’_* is the absorbance of ethanol solution; *A_s_* is the absorbance after the reaction of ABTS•+ working solution with the polyphenol extract, and *A_s’_* is the absorbance after mixing ethanol solution with the polyphenol extract.

#### 2.8.4. Hydrogen Peroxide (H_2_O_2_) Scavenging Capacity

The assay was performed based on the procedures [27], with some minor adjustments. To summarize, polyphenols (0.6 mL) were added to sodium phosphate buffer (1.5 mL, 45 mM, pH 7.4) and 400 mM H_2_O_2_ (0.9 mL). The solution was kept in a dark setting at 30 °C for 40 min, after which the absorbance was recorded at 230 nm. The results were calculated based on the following equation:

(1) Hydrogen peroxide scavenging activity (%) = [1 − (*A_s_* − *A_s’_*)/(*A_c_* − *A_c’_*)] × 100

where *A_s_* denotes the absorbance of the solution including H_2_O_2_, sodium phosphate buffer, and polyphenol extract; *A_s’_* denotes the absorbance of the sodium phosphate buffer solution; *A_c_* is the absorbance after mixing the H_2_O_2_ solution with the sodium phosphate buffer solution; *A_c’_* denotes the absorbance of the sodium phosphate buffer solution.

#### 2.8.5. Hydroxyl Radical (•OH) Scavenging Capacity

This experiment was assessed following the method described in [24]. For this assay, a combination was made by blending 100 μL of a 9 mM ferrous sulfate (FeSO_4_) solution with 1.0 mL of 9 mM salicylic acid dissolved in ethanol, along with 1 mL of an 8.8 mM H_2_O_2_ solution. After this, 100 μL of a 9 mM hydrogen peroxide solution was introduced to the mixture, which was then incubated at 37 °C for 30 min. The absorbance was recorded at 510 nm. The calculations were performed utilizing the following equation:

Hydroxyl radical scavenging activity (%) = [1 − (*A_x_* − *A_x_*_0_)]/*A*_0_ × 100

where *A_x_* is the absorbance of the solution containing polyphenol extract, FeSO_4_, H_2_O_2_, and salicylic acid–ethanol; *A_x_*_0_ is the absorbance of the solution containing polyphenol extract, FeSO_4_, H_2_O, and salicylic acid–ethanol; and *A*_0_ indicates the absorbance of the solution composing H_2_O, FeSO_4_, H_2_O_2_, polyphenol extract, and salicylic acid–ethanol.

### 2.9. Inhibition of α-Amylase, α-Glucosidase, and Lipase Activity

#### 2.9.1. α-Amylase Inhibition

This experiment was conducted based on the procedure [28]. Summarily, a volume of 0.5 mL of a-amylase solution (13 U/mL) was combined with an equal volume of SPs extract at different concentrations along with 0.1 M of a phosphate buffer solution (pH 6.9). This solution was then kept at 37 °C for 30 min. Subsequently, 0.5 mL of a 1.0% soluble starch solution was introduced, and the mixture was maintained at 37 °C for another 30 min. After incubation, 1 mL of DNS reagent was introduced; the mixture was further incubated in a boiling water bath for 5 min, then immediately cooled to room temperature with ice, followed by the addition of acarbose and water. A positive control represented absorbance measurements taken at a wavelength of 540 nm. The formula provided below was utilized to calculate the inhibitory activity:

$$\mathrm{Inhibition}\;(\%)=\left[1-\left(\frac{A_{s}-A_{s}^{\prime }}{A_{c}-A_{c}^{\prime }}\right)\right]\times 100$$

where *A_s_* denotes the absorbance of the polyphenols reaction tube composing the substrate, enzyme, SPs, and DNS; *A_s_′* refers to the absorbance of the polyphenols blank tube that included the buffer solution, substrate, SPs, and DNS; *A_c_′* represents the absorbance of the control blank tube encompassing the buffer solution, substrate and DNS; *A_c_* indicates the absorbance of the control reaction tube containing the buffer solution, enzyme, substrate, and DNS.

#### 2.9.2. α-Glucosidase (α-GLU) Inhibition

This experiment was conducted following the procedure (26), albeit with minor adjustments. In summary, 0.5 mL of an α-glucosidase solution (1 U/mL) was combined with 0.5 mL of SPs extract and 0.7 mL of phosphate buffer (0.1 M, pH 6.9). The mixture was then pre-incubated at 37 °C for 10 min. Subsequently, 0.5 mL of 5.0 mM pNPG was added to the mixture and then incubated again at 37 °C for an additional 10 min. Then, 2 mL of sodium carbonate (0.1 M) was added to halt the enzyme reaction, after which the absorbance was recorded at 540 nm. Acarbose acted as the positive control. The inhibition percentage of α-GLU activity was calculated using the equation provided:

$$\mathrm{Inhibition}\;(\%)=\left[1-\left(\frac{A_{s}-A_{s}^{\prime }}{A_{c}-A_{c}^{\prime }}\right)\right]\times 100$$

where *A_s_* denotes the absorbance value of the mixture of enzyme, pNPG, SPs, and Na_2_CO_3_; *A_s_′* refers to the absorbance value of the mixture after the buffer solution replaces the enzyme solution; *A_c_* signifies the absorbance value of the buffer solution replacing the SPs. *A_c_′* indicates the absorbance value of the buffer solution replacing the SPs and the enzyme solution.

#### 2.9.3. Lipase Inhibition

The test was performed based on the methods described in the previous method [29]. Briefly, a mixture was prepared by combining 0.04 mL of phosphate buffer (0.1 M, pH 6.9), an equal volume of the SPs extract, and 0.04 mL of lipase solution (2.5 mg/mL). This solution was incubated at 37 °C for 10 min, followed by 0.02 mL of p-Nitrophenyl Phosphate solution (pNPP, 0.01 M) was introduced. The reaction was incubated at 37 °C for an extra 15 min, after which 100 μL of anhydrous ethanol was introduced to terminate the reaction. The orlistat was included as a positive control, and the absorbance was recorded at 405 nm. The lipase inhibitory activity (%) was calculated using the formula below:

$$\mathrm{Inhibition}\;(\%)=\left[1-\left(\frac{A_{s}-A_{s}^{\prime }}{A_{c}-A_{c}^{\prime }}\right)\right]\times 100$$

where *A_s_* refers to the absorbance value of the mixture of enzyme, pNPP, and SPs; *A_s_′* refers to the absorbance value of the mixture after the buffer solution replaces the enzyme solution; *A_c_* signifies the absorbance value of the buffer solution replacing the SPs solution; *A_c_′* indicates the absorbance value of the buffer solution replacing the sample and enzyme solution.

### 2.10. Cell Culture

The cells were sourced from the Cell Resource Center of the College of Food Science and Technology, Yunnan Agricultural University. All HepG2 cells were cultured for 24 h in Dulbecco’s Modified Eagle Medium (DMEM) enriched with 10% fetal bovine serum (FBS) and 1% penicillin–streptomycin. Whereafter, the culture medium was gently aspirated, and the cells were washed softly two times with phosphate-buffered saline (PBS, 0.01 M, pH 7.4), and then, 0.25% trypsin solution was added to complete digestion (27).

### 2.11. MTT Assay

This assay was conducted to analyze cell viability following a previously established method [30], with slight adjustments. In summary, all cells were deprived of serum overnight and subsequently incubated with SP extract, which varied from 0 to 100 μg FAE/mL. This polyphenol extract was derived from samples that had undergone in vitro digestion. After this procedure, a combination of 10 μL of MTT solution (5 mg/mL) and 190 μL of PBS was introduced, while the original culture medium was carefully removed. To dissolve the formed purple formazan crystals, 100 μL of DMSO was introduced to each well. Once the crystals were fully dissolved, the absorbance was recorded at 490 nm.

### 2.12. Assessment of Oxidative Stress Markers

The determination of intracellular oxidative stress parameters was conducted following the previous method [31]. The fluorescent probe 2′,7′-dichlorofluorescin diacetate (DCFH-DA) was employed to evaluate the quantities of ROS. In brief, HepG2 cells (2 × 10^4^ cells/well) were plated in a 96-well plate and incubated for 24 h. After this, they underwent serum starvation overnight. Following this, the cells were exposed to non-toxic polyphenol extract for 24 h, after which they were treated with H_2_O_2_ (800 μM) and further incubated for an extra 6 h. Post-incubation, the cells were washed twice with PBS and then treated with 10 μM DCFH-DA at 37 °C in the dark for 30 min. The fluorescence intensity was recorded with excitation and emission wavelengths set at 488 nm and 525 nm, respectively.

The cells (2 × 10^5^ cells per well) were grown in 6-well plates and treated according to previously established methods [30]. The antioxidant factors assessed included MDA, GPx, SOD, CAT, GSH, and T-AOC, which were measured with corresponding kits.

### 2.13. Statistical Analysis

Alongside the roasted HB, raw HB was included as a control group to establish a baseline for comparison. The raw HB and the roasted HB underwent the same analytical procedures to guarantee the validity and reliability of the experimental outcomes. To assure consistent findings, these experiments were performed in triplicate, with the results expressed as mean ± standard deviation (mean ± SD), which effectively represents the variability present in the data. Data analysis was executed using SPSS version 26.0 to evaluate the significance of differences among two or more groups. A significance threshold of *p* < 0.05 was set to denote statistically significant differences. For graphical representation, both bar and line graphs were created utilizing GraphPad Prism 8.0 software. To investigate the differences among the groups, we employed multiple principal component analysis (PCA) through SIMICA 14 software. Furthermore, correlation analyses were conducted using Origin 7.0.

## 3. Results and Discussion

### 3.1. Morphological Property Analysis

**Figure 1:**
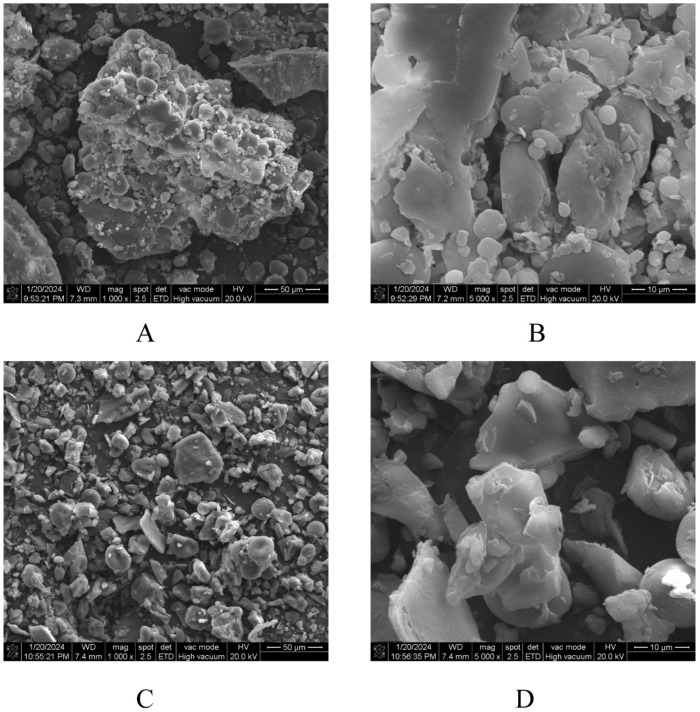
The SEM photos of different samples: (**A**,**B**) raw HB, (**C**,**D**) roasted HB. The SEM photos of the samples were recorded at 1000× and 5000× magnification. Raw HB: raw highland barley; roasted HB: roasted highland barley.

To investigate the release mechanism of polyphenols, the impact of roasting on the surface structure of HB was analyzed in Figure 1. The microstructure of raw HB displayed large, chunky, and spherical entities, likely representing residual protein aggregates and small starch granules [32]. In contrast, roasted HB exhibited a more porous structure characterized by smaller fragments, a rougher surface, a reduced number of white spherical particles, and significant particle separation. This alteration may be attributed to the gelatinization of starch and the denaturation of protein induced by the roasting [33]. Furthermore, the surface particles of roasted HB were more loosely and uniformly distributed, which enhanced the exposure of the internal structure. This observation suggested that roasting may disrupt the tightly packed fibrous network of cell walls, thereby creating regions that facilitate the release of polyphenols. These findings align with the results reported by previous research [33].

**Figure 2:**
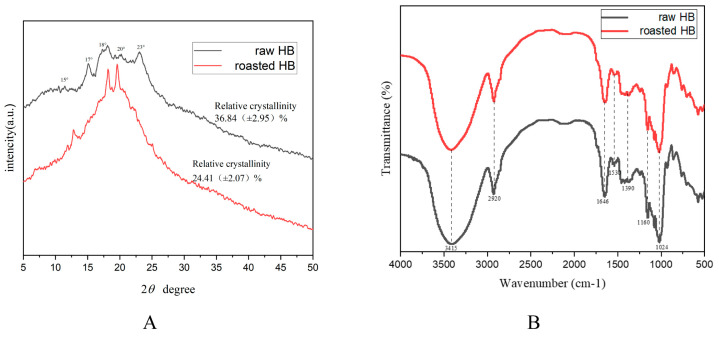
XRD spectra (**A**) and FT-IR spectra (**B**) of different samples. raw HB: raw highland barley; roasted HB: roasted highland barley.

To further investigate the change in crystalline structure, the X-ray diffraction (XRD) patterns of HB before and after roasting were analyzed. As illustrated in Figure 2A, raw HB exhibited a typical A-type crystalline structure, characterized by main peaks at 15°, 17°, 18°, and 23° [34]. In contrast, after roasting, the peaks at 17° and 23° were significantly suppressed or disappeared, while a sharp crystalline peak emerged at 18° and 20° with enhanced absorbance. The crystallinity of the samples was estimated using Jade software 6.0, revealing relative crystallinity values of 36.84% for raw HB and 24.41% for roasted HB. The significant decrease in relative crystallinity post-roasting indicated damage to the semi-crystalline starch structure [34]. Following roasting, the samples displayed features characteristic of both A-type and V-type crystalline structures, with a peak emerging at approximately 18° and 20° (2θ), suggesting the formation of starch double helices or amylose–lipid complexes during the roasting process [35]. Compared to raw HB, the peak intensity of 18° and 20° of roasted HB flour increased markedly. The XRD results indicated that roasting could disrupt the crystalline regions of cellulose and hemicellulose, facilitating the release of a substantial amount of polyphenols. These findings were consistent with results obtained from scanning electron microscopy and antioxidant activity assays.

The difference in functional groups and chemical bonds among various samples was characterized using FT-IR spectroscopy; the result is shown in Figure 2B. The typical infrared absorption peaks of the samples were observed at 3415, 2920, 1646, 1530, 1390, 1160, and 1024 cm^−1^. Notably, the broad and intense absorption peak at 3415 cm^−1^ in both raw and roasted HB is associated with the bending and stretching vibrations of O–H groups, which are commonly found in cellulose and hemicellulose components [36]. In comparison to raw HB, the peak at 3415 cm^−1^ in roasted HB exhibited a narrower and reduced intensity, suggesting that roasting may disrupt the cellulose and hemicellulose structure, thereby facilitating the release of polyphenols [36]. The absorption peak at approximately 2920 cm^−1^ is attributed to the stretching vibrations of –CH_2_ groups [36]. The peak at 1646 cm^−1^ is associated with the C=O stretching vibrations of –CHO groups and/or the NH groups of proteins [36]. The characteristic absorption peak of aromatic benzene rings in lignin was observed at 1530 cm^−1^ [37]. The absorption peaks within the range of 1390 to 1160 cm^−1^ in both raw and roasted HB were primarily attributed to the bending vibrations of C–H groups or the stretching vibrations of C–N groups, indicating the presence of protein. At approximately 1024 cm^−1^, a distinct peak found in the sample was attributed to the stretching vibration of the C–O–C groups, which is related to the presence of polysaccharide components [37].

### 3.2. The Change in TPC and TFC Before and After In Vitro Digestion

**Figure 3:**
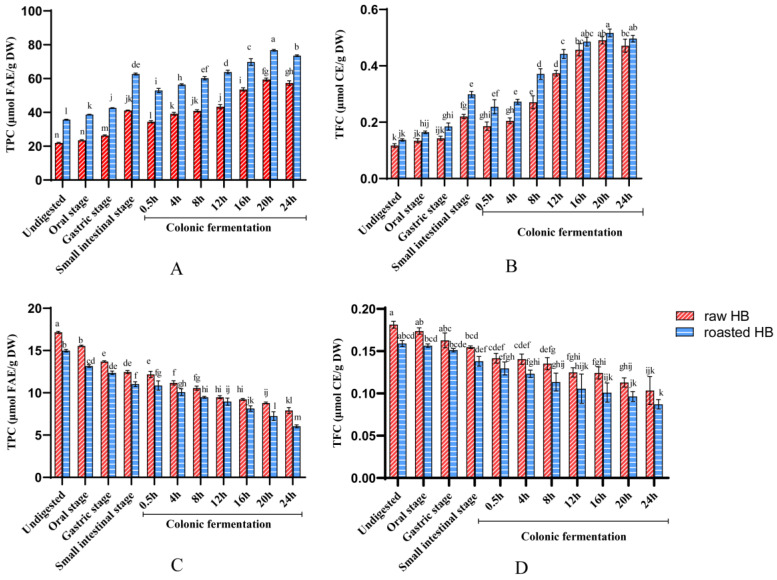
Influence of in vitro digestion on TPC and TFC Content in soluble fraction (**A**,**B**) and insoluble bound fraction (**C**,**D**) of raw and roasted HB Flour. Different lowercase letters represent a significant difference between raw HB flour and roasted HB flour at different phases (*p* < 0.05).

The TPC and TFC of raw and roasted HB subjected to in vitro gastrointestinal digestion and colonic fermentation are shown in Figure 3. For the soluble fraction, the change in TPC and TFC from raw HB during gastrointestinal digestion occurred in the following order: intestinal > gastric > oral > undigested. Notably, compared to the undigested phase, the TPC of raw HB exhibited a significant increase during both the gastric and intestinal phases, with increments of 19.86% and 87.29%, respectively. Concurrently, TFC showed a marked increase in the intestinal phase (*p* < 0.05), escalating from 0.12 ± 0.01 μmol CE/g DW to 0.22 ± 0.01 μmol CE/g DW. The TPC and TFC in roasted HB displayed a similar trend to those in raw HB, with the intestinal phase showing the highest values, followed by the gastric, oral, and undigested phases. Compared to the undigested stage, the TPC of the roasted sample increased by 8.59%, 19.43%, and 75.81% in the oral, gastric, and intestinal phases, respectively. Similarly, the TFC exhibited increments of 14.28%, 28.57%, and 114.29% in these stages. Several studies have confirmed the enhancement in TPC and TFC during gastrointestinal digestion, as observed in sweet potato leaves [38] and wheat bran [39]. This phenomenon may be attributed to the action of digestive enzymes or a change in pH that promotes the release of IBPs. The observed variations in TPC and TFC concerning IBPs further support this perspective [40]. After undergoing colonic fermentation, the TPC and TFC of the samples displayed a pattern marked by an initial rise, which was then succeeded by a decline, peaking at colonic fermentation for 20 h. These fluctuations in TPC and TFC during colonic fermentation may be attributed to the metabolic and degradative action of the microbial environment in the gut. Our research results align with the conclusion drawn from a previous study [39].

For IBPs, the TPC and TFC in both samples decreased progressively during the in vitro digestion, ultimately reaching their minimum level following colonic fermentation for 24 h. We hypothesized that during in vitro digestion, the majority of IBPs are released from their complex structures due to the effects of pH, digestive enzymes, or microbial activity, which results in elevated TPC and TFC levels in the soluble fraction [39,41].

Interestingly, the soluble fraction obtained from roasted HB exhibited a markedly elevated TPC when compared to the raw HB during the in vitro digestion process (*p* < 0.05). In contrast, the insoluble bound fraction showed an inverse trend. Food processing techniques can prompt the release or breakdown of polyphenols. Numerous earlier investigations have shown that heat treatments have a beneficial impact on the liberation of polyphenols during in vitro digestion [13,42]. For example, one study indicated that various heat processing techniques, such as steaming, baking, boiling, and microwaving, considerably raised the level of polyphenols in sweet potatoes [13]. This suggests that thermal treatments can facilitate the release of polyphenols by altering their structure or the manner in which they are bound to the food matrix, thereby enhancing their bioaccessibility during in vitro digestion.

### 3.3. Antioxidant Capacity of Polyphenols

**Figure 4:**
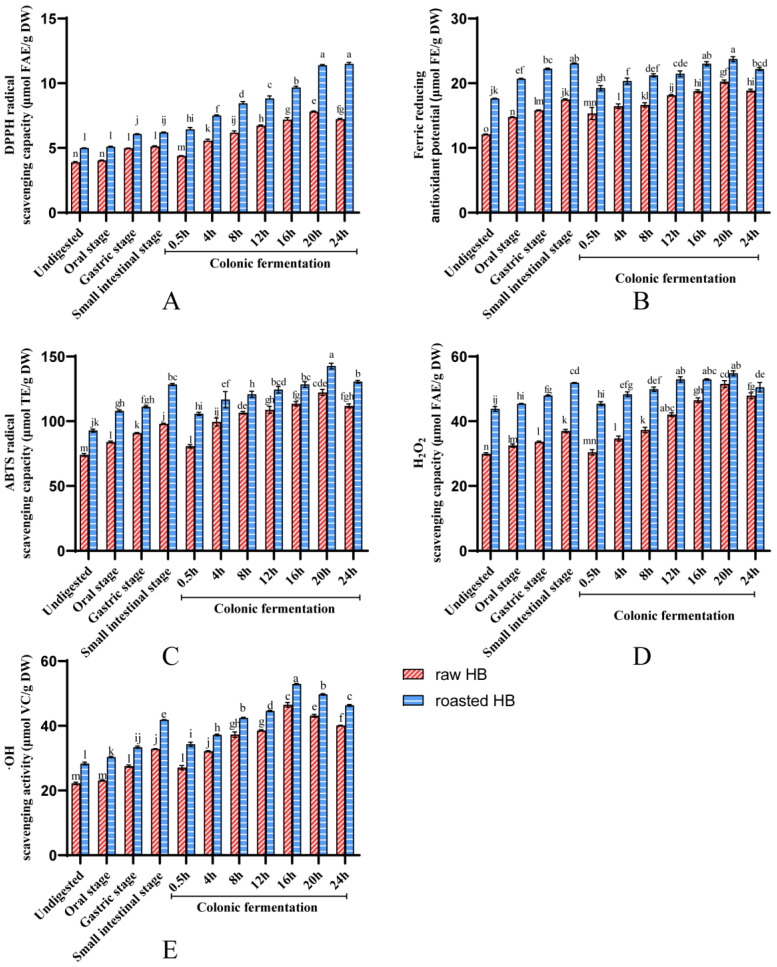
For soluble fraction, (**A**) DPPH radical scavenging activity, (**B**) Ferric-ion reducing capacity, (**C**) ABTS radical scavenging activity, (**D**) H_2_O_2_ scavenging activity, (**E**) Hydroxyl radical scavenging activity. Different lowercase letters represent a significant difference between raw HB flour and roasted HB flour at different phases (*p* < 0.05).

**Figure 5:**
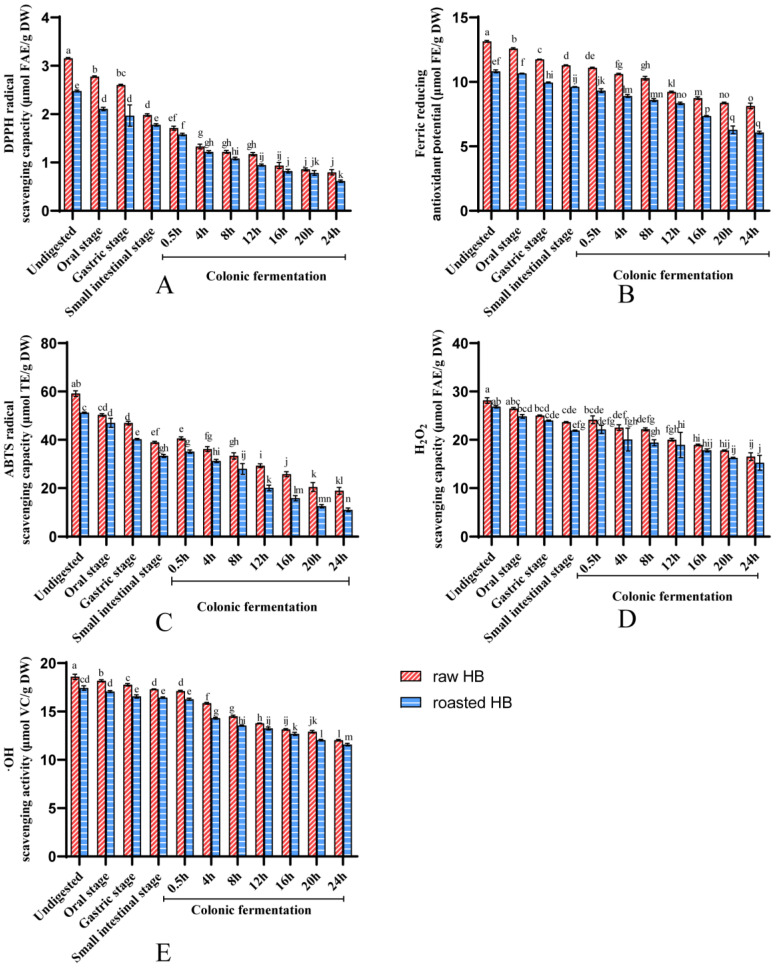
For insoluble bound fraction, (**A**) DPPH radical scavenging activity, (**B**) Ferric-ion reducing capacity, (**C**) ABTS radical scavenging activity, (**D**) H_2_O_2_ scavenging activity, (**E**) Hydroxyl radical scavenging activity. Different lowercase letters represent a significant difference between raw HB flour and roasted HB flour at different phases (*p* < 0.05).

Polyphenols are the primary bioactive compounds in HB, exhibiting strong free radical scavenging and antioxidant activities [43]. The antioxidant property of the obtained polyphenols of HB throughout in vitro digestion was measured employing the following methods: DPPH, FRAP, ABTS, H_2_O_2_, and •OH. The results demonstrated that the antioxidant capacity of SPs and IBPs was illustrated in Figure 4 and Figure 5, respectively. In the gastric and intestinal phases, the soluble fraction had significantly elevated antioxidant activity in raw and roasted HB compared with that in the undigested stage (*p* < 0.05). Compared with the undigested stage, the increase in antioxidant indexes (DPPH, FRAP, ABTS, H_2_O_2_, and •OH) of raw HB in the intestinal stage was 31.36%, 44.50%, 32.48%, 23.65%, and 47.75%, respectively. At the same time, for the roasted HB, the enhancement was 24.09%, 30.78%, 38.48%, 18.34%, and 47.54%, respectively. Several studies have confirmed the enhancement of the antioxidant capacity of SPs during the gastrointestinal process. Chait et al. reported that during the in vitro simulation of gastrointestinal digestion of carob pods, the release of carob polyphenols was enhanced, which, in turn, led to an increase in antioxidant activity. In another study on in vitro gastrointestinal digestion of gastrodia, the polyphenol content in digested samples was 1.03 times higher than in undigested samples, and their DPPH free radical scavenging activity increased by 43.10%. Conversely, the antioxidant capacity of the IBPs typically exhibited a downward performance during the gastrointestinal process [43]. This decline may result from the release of IBPs in the gastrointestinal tract, which converts them into the soluble fraction, leading to a decrease in the antioxidant activity of the insoluble bound fraction.

The antioxidant property of the SPs in raw and roasted HB (except for the DPPH in roasted HB) demonstrated an initial rise, which was later followed by a decrease (Figure 4) after colonic fermentation. Conversely, the antioxidant properties of the IBPs in these samples displayed a consistent downward trend during colonic fermentation (Figure 5). Importantly, these findings correspond with the variations in TPC and TFC noted in both SPs and IBPs. The literature has reported comparable trends in SPs, revealing an initial rise in antioxidant capacity, which is subsequently followed by a decline during colonic fermentation. This phenomenon is illustrated by the DPPH and ABTS measurements recorded in carrots throughout the process of colonic fermentation [20]. Similarly, a study reported the highest DPPH and FRAP values for mung bean coats after colonic fermentation for 12 h. Concurrently, the antioxidant property of the sample submitted to colonic fermentation for 24 h decreased, which may be associated with the reduction in TPC [44]. This suggests that the early release of polyphenols by the gut microbiota is subject to slow degradation and/or breakdown.

It was observed that at each phase of the in vitro digestion procedure, the soluble fraction of raw HB showed lower antioxidant capacity compared to that of roasted HB (Figure 4). Conversely, the insoluble bound fraction from raw HB demonstrated stronger antioxidant capacities than its roasted counterparts (Figure 5). Numerous studies have established a positive correlation between TPC and antioxidant activity (16,40,41). We proposed that the improved antioxidant capacity observed in the soluble fraction of roasted HB during in vitro digestion can be linked to the structural alteration of polyphenols associated with fibers and protein as a consequence of the roasting process. This modification likely facilitates a greater release of IBPs throughout digestion.

### 3.4. Bioaccessibility of Polyphenols

Bioaccessibility of polyphenols refers to the quantity of a compound that is released from the gastrointestinal tract and is available for absorption. In contrast, bioavailability refers to the extent to which a compound enters the systemic circulation and exerts its biological activity. Polyphenols become bioaccessible and can exert beneficial health-promoting effects only when they are released from food [45,46]. As shown in Table 1 and Table 2, the TPC, TFC, DPPH, FRAP, ABTS, H_2_O_2_, and •OH bioaccessibility of roasted HB in the gastrointestinal tract increased by 52.53%, 35.74%, 21.10%, 32.06%, 40.30%, 27.41%, and 31.11%, respectively, compared to raw HB, while in the lower digestive stage, these values increased by 28.29%, 5.35%, 59.28%, 17.53%, 5.42%, 15.61%, and 16.68%, respectively. Earlier research has indicated that the absorption of dietary polyphenols in the human body is limited, as certain polyphenols, together with indigestible carbohydrates and various plant materials (including lignin and proteins), hinder the processes of digestion and absorption [46]. However, certain processing methods, particularly thermal treatments, can either enhance or reduce the bioaccessibility of polyphenols. Compared to raw HB, the bioaccessibility of polyphenols in roasted HB significantly increased (*p* < 0.05). In summary, our findings support the notion that roasting can improve the bioaccessibility of polyphenols during in vitro digestion, with similarities to earlier research [47].

### 3.5. Quantification of Phenolic Compounds Using HPLC

In this research, it was discovered that the soluble fraction of the samples included five types of hydroxybenzoic acids, five kinds of hydroxycinnamic acids, and eight flavonoids (Table 3). After undergoing in vitro digestion, the level of hydroxybenzoic acids and flavonoids in raw HB diminished during gastrointestinal digestion; however, they showed a significant increase following colonic fermentation when compared to the samples that had not been digested. In contrast, the level of hydroxybenzoic acids and flavonoids in roasted HB was markedly enhanced after in vitro digestion. For hydroxycinnamic acids, the content in raw HB significantly increased after in vitro digestion. In roasted HB, the level of hydroxycinnamic acids showed a considerable rise after gastrointestinal digestion, yet they experienced a significant reduction after colonic fermentation. Overall, the highest content of SPs was observed after colonic fermentation in raw and roasted HB, indicating that the colonic environment positively influenced the release of polyphenols. Additionally, during the undigested and gastrointestinal digestion stages, the TPC of SPs in roasted HB was found to be higher than that in raw HB, indicating that roasting may facilitate the release of polyphenols. Notably, significant disparities in the concentration of specific compounds were observed between the raw and roasted HB at various stages of digestion. In the case of raw HB, the predominant compounds at the undigested, gastrointestinal digestion, and colonic fermentation stages were (−)-epigallocatechin, quercetin, and gallic acid, respectively. Conversely, for roasted HB, chlorogenic acid was the most abundant compound at both the undigested and gastrointestinal digestion stages, while (−)-epigallocatechin was the most prevalent at the colonic fermentation stage. These differences are likely attributable to the transformation of polyphenols during digestion. For instance, the increase in the content of certain phenolic acids in the colonic stage, like ferulic acid, may be caused by the metabolites of chlorogenic acid [48]. Furthermore, during colonic fermentation, gut microbiota can release bound polyphenols from the matrix and further metabolize them into free polyphenols, thereby increasing their concentration [46].

In the IBPs, we observed a minimal amount of hydroxycinnamic acids and flavonoids across various stages of digestion (Table 4). In raw HB, the concentration order of these compounds is intestinal > undigested > colonic. Conversely, roasted HB exhibited a pattern where the undigested phase had a higher level than the gastrointestinal phase, which, in turn, surpassed the colonic phase. Overall, the TPC in the insoluble bound fraction of roasted HB was significantly lower than that of raw HB after digestion (*p* < 0.05). This suggests that the roasting may promote the release of IBPs. The disruption of plant cell wall structures, including cellulose, hemicellulose, and lignin, during roasting, facilitates the breaking of ester and ether bonds between polyphenols and polysaccharides or proteins in the cell wall, thereby releasing the IBPs into a free form [12,47].

### 3.6. Principal Component Analysis

**Figure 6:**
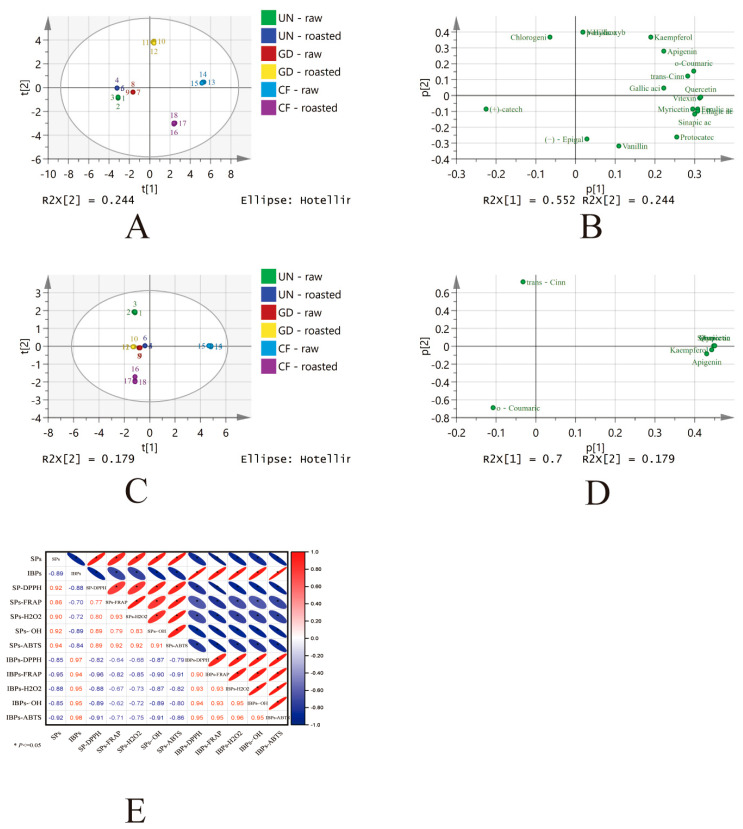
Score and loading diagrams of soluble (**A**,**B**) and insoluble fractions (**C**,**D**) in raw and roasted HB across digestion phases. Polyphenol-antioxidant correlations in HB (**E**). UN-raw: undigested raw HB flour; UN-roasted: undigested roasted HB flour; GD-raw: gastrointestinal digested raw HB flour; GD-roasted: gastrointestinal digested roasted HB flour; CF-raw: colonic fermented raw HB flour; CF-roasted: colonic fermented roasted HB flour.

The loading and score plots are illustrated in Figure 6A–D. The principal components PC1 and PC2 of the soluble fraction accounted for 55.2% and 24.4% of the variance, respectively. In contrast, for the insoluble bound fraction, PC1 and PC2 explained 70.0% and 17.9% of the variance, respectively. The cumulative variance contribution for both soluble and insoluble bound fractions reached 95.0%. Significant differences were observed between raw and roasted HB at various digestion stages. In the soluble fraction, the primary contributors to PC1 included vitexin, quercetin, ferulic acid, myricetin, ellagic acid, and sinapic acid. This analysis confirms that roasting induced changes in the polyphenols.

### 3.7. Correlation Between TPC and Antioxidant Activity

The antioxidant activity of plant materials is primarily influenced by polyphenol content. Generally, a pronounced correlation is observed between TPC and antioxidant activity [39,41,47]. This study analyzed the correlation between TPC and antioxidant activity in raw and roasted HB across various in vitro digestion stages. As depicted in Figure 6E, a notable positive correlation was observed between antioxidant activity and TPC (*p* < 0.05). These findings suggest that the antioxidant activity of HB samples largely depends on their TPC. Furthermore, a strong negative correlation was noted between SPs and IBPs (*p* < 0.05). The results indicate that the antioxidant activity of HB samples is associated with TPC, and the digestion process facilitates the release of IBPs.

### 3.8. Inhibition of Enzyme Activity

**Figure 7:**
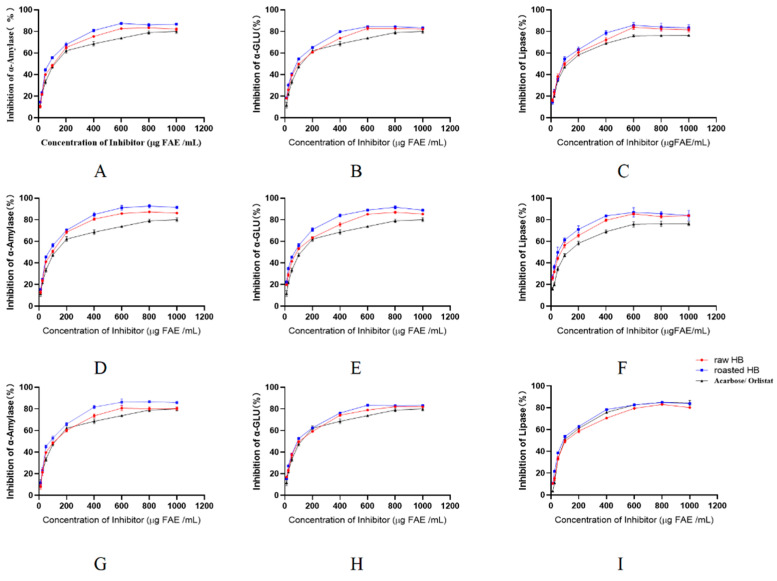
Inhibitory effects of HB polyphenol extracts on α-amylase, α-glucosidase, and lipase at different stages: (**A**–**C**) undigested, (**D**–**F**) gastrointestinal digested, and (**G**–**I**) colonic fermented.

As illustrated in Figure 7, within the concentration range of 400~1000 μg FAE/mL, the extract of raw and roasted HB at diverse in vitro digestion stages (except for raw HB at the colonic fermentation, Figure 7I) had stronger inhibitory effects on α-amylase, α-glucosidase, and lipase than acarbose and oristat. It has been reported that dietary polyphenols can alleviate postprandial hyperglycemia and reduce the absorption of fats by inhibiting key digestive enzymes (α-amylase, α-glucosidase, and lipase) (46). This implies that polyphenols may serve as potential agents for alleviating the burden of diabetes and obesity. Our experimental results were expressed as half-maximal inhibitory concentration (IC_50_) values, with acarbose and orlistat serving as positive controls (Table 5). The inhibitory activity of the polyphenols of raw and roasted HB at different digestive stages against the three enzymes showed the same sequence: gastrointestinal > undigested > colonic fermentation. Furthermore, the inhibitory activity of the polyphenols from roasted HB was significantly greater than that of the raw HB at every digestion stage (*p* < 0.05) (Table 5), suggesting that polyphenol extract from roasted HB enhanced the potential for alleviating postprandial hyperglycemia and managing obesity.

### 3.9. Cell Viability

**Figure 8:**
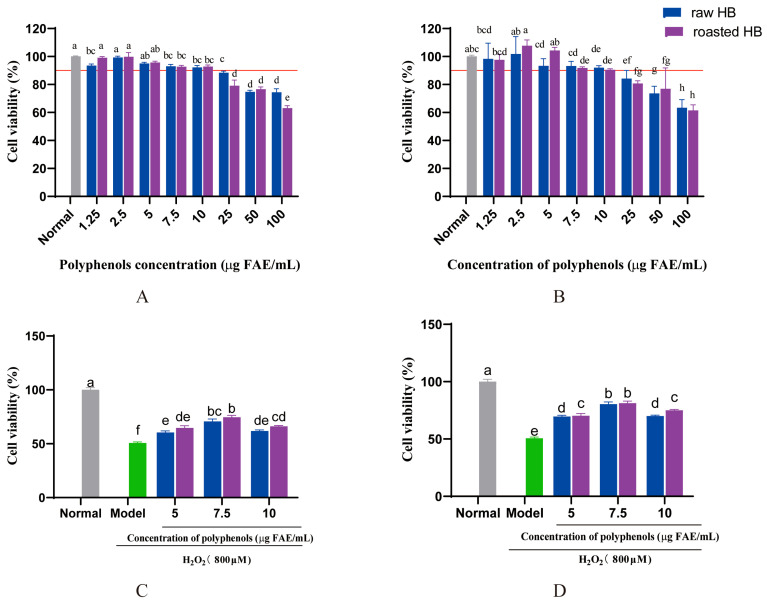
Cytoprotective effects of polyphenol extract from HB after in vitro gastrointestinal digestion (**A**) and colonic fermentation for 24 h (**B**). Cell viability of H_2_O_2-_induced oxidative damage in HepG2 cells treated with polyphenol extract from HB after in vitro gastrointestinal digestion (**C**) and colonic fermentation for 24 h (**D**).  The data marked by different letters are significantly different (*p* < 0.05). All values are means ± SD (*n* = 3).

The cytotoxic effect of digested HB polyphenols was evaluated on HepG2 cells using the MTT assay. Cells were exposed to digested HB polyphenols at concentrations varying from 0 to 100 μg/mL for 24 h (Figure 8). The cytotoxicity test demonstrated that cell viability remained above 90% when the concentration of HB polyphenols was below 10 mg/mL, indicating a lack of cytotoxicity toward HepG2 cells (Figure 8A,B).

To investigate the protective effect of digested SPs extracted from HB on H_2_O_2_-induced oxidative stress damage, the viability of HepG2 cells was assessed. As depicted in Figure 8C,D, compared with the normal group (without H_2_O_2_ treatment), the H_2_O_2_-treated group (model), cell viability significantly decreased to 50.67%, with *p* < 0.05. Interestingly, the viability of cells subjected to H_2_O_2_ was substantially enhanced by the digested SP extracts, *p* < 0.05. Moreover, the cells incubated with digested roasted HB extract exhibited higher viability than those treated with an equivalent concentration of raw HB extract. A previous study has demonstrated that raisin polyphenols can significantly enhance the viability of HepG2 cells damaged by oxidative stress, which is similar to our findings [49].

### 3.10. Impact of Polyphenol Extract on H_2_O_2_-Induced Oxidative Stress in HepG2 Cells

ROS are widely acknowledged for their pivotal role in the modulation of various metabolic disorders associated with multiple diseases [50]. The inhibition of ROS generation and the enhancement of antioxidant defenses have been established as promising therapeutic strategies for conditions related to oxidative stress. Hydrogen peroxide (H_2_O_2_), a primary contributor to ROS production, is involved in numerous physiological and pathological processes within cells, resulting in oxidative damage. Consequently, H_2_O_2_ is frequently utilized as an inducer of oxidative stress injury [51].

**Figure 9:**
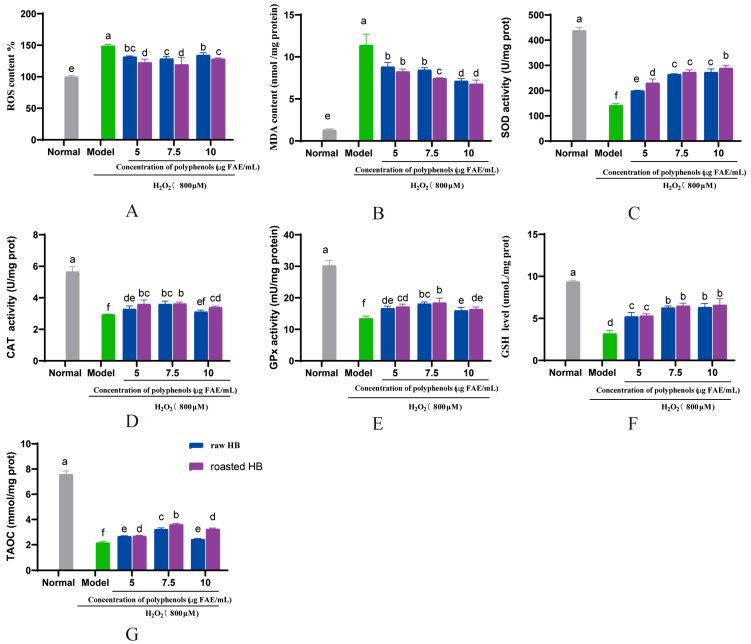
Effect of HB after in vitro gastrointestinal digestion on H_2_O_2_-induced oxidative damage in HepG_2_ cells. (**A**) ROS. (**B**) MDA. (**C**) SOD. (**D**) CAT. (**E**) GSH. (**F**) GPx. (**G**) T-AOC. Different letters show significant differences at *p* < 0.05.

**Figure 10:**
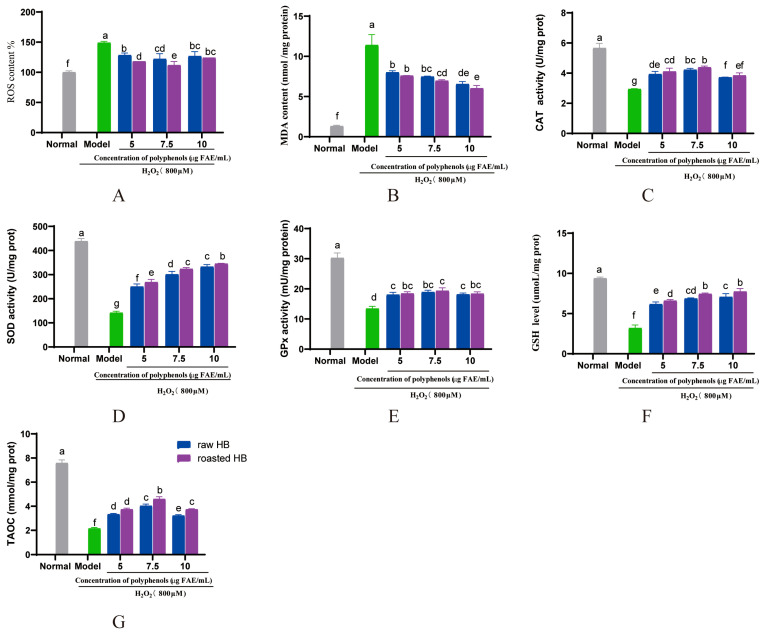
Effect of HB after colonic fermentation for 24 h on H_2_O_2_-induced oxidative damage in HepG_2_ cells. (**A**) ROS. (**B**) MDA. (**C**) SOD. (**D**) CAT. (**E**) GSH. (**F**) GPx. (**G**) T-AOC.  Different letters show significant differences at *p* < 0.05.

In this investigation, we noted a marked decrease in ROS production in HepG2 cells that were pre-treated with digested HB polyphenols, in contrast to the model group (*p* < 0.05) (Figure 9A and Figure 10A). A previous study demonstrated that digested bayberry extract can reduce ROS production in HepG2 cells [52]. Antioxidant enzymes, such as SOD, CAT, and GPx, represent the first line of defense against oxidative stress. Recent reports indicated that wild artichoke leaf extract can prevent oxidative stress by enhancing the intracellular activity of antioxidant enzymes and reducing ROS formation [53]. Furthermore, the ethanol extract from mulberry leaves showed a promising effect in alleviating oxidative stress-related damage in HepG2 cells [54]. Our results align with these findings, suggesting that digested HB polyphenols may confer a protective effect against oxidative stress by lowering the level of ROS and MDA while enhancing the activity and expression of SOD, CAT, GPx, GSH, and T-AOC (Figure 9 and Figure 10). Notably, our study found that the digested roasted HB extract exhibited a more pronounced protective effect against H_2_O_2_-induced oxidative stress damage compared to the raw HB.

## 4. Conclusions

This study investigated the impact of roasting on the release of polyphenols from HB, as well as the bioaccessibility and bioactivity of these compounds during in vitro digestion. Morphological property analysis indicated that roasting can disrupt the tightly packed cellulose and protein networks within the cell walls, thereby facilitating the release of IBPs. During in vitro digestion, polyphenols derived from roasted HB demonstrated improved bioaccessibility and bioactivity. Furthermore, in comparison with raw HB, polyphenol extract from roasted HB, subjected to gastrointestinal digestion and colonic fermentation for 24 h, more effectively alleviated H_2_O_2_-induced oxidative stress in HepG2 cells. These discoveries lay a strong groundwork for the development of HB products with improved bioactivity and potential health benefits. However, the bioavailability of polyphenols from roasted HB warrants further investigation.

## Figures and Tables

**Table 1:** Bioaccessible polyphenols after in vitro gastrointestinal digestion.

	Indigestion		Gastrointestinal Digestion		Bioaccessibility(%)	
	Raw HB	Roasted HB	Raw HB	Roasted HB	Raw HB	Roasted HB
TPC (µmol FAE/g DW)	21.95 ± 0.32 ^b^	35.67 ± 0.32 ^a^	41.24 ± 0.27 ^b^	62.78 ± 0.56 ^a^	187.28 ^b^	285.65 ^a^
TFC (µmol CE/g DW)	0.12 ± 0.01 ^b^	0.14 ± 0.00 ^a^	0.22 ± 0.01 ^b^	0.30 ± 0.01 ^a^	188.13 ^b^	255.36 ^a^
DPPH (μmol FAE/g DW)	3.88 ± 0.05 ^b^	4.99 ± 0.01 ^a^	5.14 ± 0.06 ^b^	6.20 ± 0.04 ^a^	131.65 ^b^	159.43 ^a^
FRAP (μmol FE/g DW)	12.09 ± 0.08 ^b^	17.64 ± 0.04 ^a^	17.53 ± 0.12 ^b^	23.07 ± 0.06 ^a^	144.50 ^b^	190.83 ^a^
ABTS (μmol TE/g DW)	29.90 ± 0.37 ^b^	43.83 ± 0.74 ^a^	37.17 ± 0.52 ^b^	51.81 ± 0.11 ^a^	123.63 ^b^	173.45 ^a^
H_2_O_2_ (μmol FAE/g DW)	22.22 ± 0.38 ^b^	28.35 ± 0.41 ^a^	32.89 ± 0.1 ^b^	41.82 ± 0.08 ^a^	147.76 ^b^	188.26 ^a^
•OH (μmol VC/g DW)	73.95 ± 1.13 ^b^	92.76 ± 1.16 ^a^	97.76 ± 0.56 ^b^	128.35 ± 0.71 ^a^	132.48 ^b^	173.69 ^a^

The letters denote significant differences between the two sample groups, with *p* < 0.05 based on the Student *t*-test.

**Table 2:** Bioaccessible polyphenols after in vitro colonic fermentation.

	Colonic Fermentation for 0.5 h		Colonic Fermentation for 24 h		Bioaccessibility(%)	
	Raw HB	Roasted HB	Raw HB	Roasted HB	Raw HB	Roasted HB
TPC (µmol FAE/g DW)	34.46 ± 0.7 ^b^	52.95 ± 1.25 ^a^	57.38 ± 1.33 ^b^	73.55 ± 0.52 ^a^	166.51 ^b^	213.41 ^a^
TFC (µmol CE/g DW)	0.19 ± 0.01 ^a^	0.25 ± 0.03 ^a^	0.47 ± 0.02 ^b^	0.50 ± 0.01 ^a^	253.53 ^a^	267.09 ^a^
DPPH (μmol FAE/g DW)	4.38 ± 0.03 ^b^	6.43 ± 0.15 ^a^	7.22 ± 0.06 ^b^	11.50 ± 0.10 ^a^	164.97 ^b^	262.77 ^a^
FRAP (μmol FE/g DW)	15.32 ± 0.93 ^b^	19.24 ± 0.4 ^a^	18.87 ± 0.21 ^b^	22.18 ± 0.22 ^a^	123.16 ^b^	144.75 ^a^
ABTS (μmol TE/g DW)	30.39 ± 0.83 ^b^	45.42 ± 0.61 ^a^	47.89 ± 0.89 ^b^	50.48 ± 1.47 ^a^	157.56 ^b^	166.10 ^a^
H_2_O_2_ (μmol FAE/g DW)	27.08 ± 0.67 ^b^	34.27 ± 0.66 ^a^	40.06 ± 0.13 ^b^	46.31 ± 0.28 ^a^	147.94 ^b^	171.03 ^a^
•OH (μmol VC/g DW)	80.65 ± 1.28 ^b^	105.70 ± 1.29 ^a^	111.87 ± 1.62 ^b^	130.54 ± 1.05 ^a^	138.72 ^b^	161.86 ^a^

The letters denote significant differences between the two sample groups, with *p* < 0.05 based on the Student *t*-test.

**Table 3:** Identification of the polyphenols from SPs in the extract of HB during in vitro digestion.

Identification	Indigestion		Gastrointestinal Digestion		Colonic Fermentation	
	Raw HB (μg/g DW)	Roasted HB (μg/g DW)	Raw HB (μg/g DW)	Roasted HB (μg/g DW)	Raw HB (μg/g DW)	Roasted HB (μg/g DW)
Hydroxybenzoic acids						
Gallic acid	6.92 ± 0.23 ^c^	32.54 ± 0.66 ^b^	ND	ND	220.10 ± 1.84 ^a^	ND
Protocatechuic acid	ND	ND	ND	ND	37.30 ± 1.21	53.75 ± 0.46
*p*-Hydroxybenzoic acid	ND	ND	ND	25.21 ± 0.81	ND	ND
Vanillin	ND	ND	ND	ND	ND	3.87 ± 0.32
Vanillic acid	ND	ND	ND	29.72 ± 0.96	ND	ND
Total	6.92 ± 0.23 ^e^	32.54 ± 0.66 ^d^	ND	54.92 ± 1.36 ^c^	257.40 ± 1.53 ^a^	57.62 ± 0.44 ^b^
Hydroxycinnamic acid						
Chlorogenic acid	ND	114.58 ± 1.40 ^b^	ND	153.86 ± 1.37 ^a^	14.31 ± 0.22 ^c^	ND
Ferulic acid	6.66 ± 0.48 ^d^	4.19 ± 0.66 ^e^	6.45 ± 0.04 ^d^	9.25 ± 0.15 ^c^	25.52 ± 0.30 ^a^	18.21 ± 0.60 ^b^
Sinapic acid	5.66 ± 0.28 ^c^	ND	17.13 ± 0.23 ^b^	17.59 ± 0.52 ^b^	41.46 ± 1.26 ^a^	40.34 ± 1.07 ^a^
*trans*-Cinnamic acid	1.90 ± 0.02 ^e^	1.48 ± 0.07 ^f^	3.03 ± 0.00 ^d^	5.24 ± 0.11 ^b^	11.97 ± 0.15 ^a^	3.80 ± 0.25 ^c^
*o*-Coumaric acid	3.32 ± 0.13 ^e^	3.06 ± 0.13 ^e^	8.64 ± 0.07 ^d^	17.60 ± 0.11 ^b^	23.80 ± 0.37 ^a^	14.04 ± 0.21 ^c^
Total	17.55 ± 0.53 ^f^	123.32 ± 1.98 ^b^	35.26 ± 0.19 ^e^	203.54 ± 1.54 ^a^	117.06 ± 1.76 ^c^	76.38 ± 1.98 ^d^
Flavonoids						
(−)-Epigallocatechin	128.89 ± 2.36 ^b^	64.10 ± 2.19 ^d^	ND	80.40 ± 2.34 ^c^	ND	310.34 ± 1.82 ^a^
Epicatechin	33.27 ± 0.99	22.73 ± 1.20	ND	ND	ND	ND
vitexin	ND	8.95 ± 0.32 ^e^	15.37 ± 0.38 ^d^	26.21 ± 0.76 ^c^	56.45 ± 1.26 ^a^	42.65 ± 0.46 ^b^
Ellagic acid	9.81 ± 0.77 ^e^	8.12 ± 0.38 ^f^	21.77 ± 0.15 ^d^	36.90 ± 0.55 ^c^	144.51 ± 0.50 ^a^	105.78 ± 0.07 ^b^
Myricetin	18.34 ± 0.63 ^e^	16.24 ± 0.45 ^e^	21.65 ± 0.73 ^d^	83.49 ± 1.03 ^c^	145.93 ± 1.31 ^b^	163.23 ± 0.67 ^a^
Quercetin	14.94 ± 1.11 ^e^	9.60 ± 0.42 ^f^	41.93 ± 0.26 ^d^	55.50 ± 1.73 ^c^	140.77 ± 1.99 ^a^	85.61 ± 0.87 ^b^
Kaempferol	7.59 ± 0.39 ^e^	5.25 ± 1.09 ^f^	13.51 ± 0.11 ^c^	47.61 ± 0.93 ^a^	35.01 ± 0.81 ^b^	13.32 ± 0.56 ^c^
Apigenin	11.19 ± 0.22 ^c^	5.55 ± 0.40 ^d^	ND	33.17 ± 0.06 ^b^	45.99 ± 0.54 ^a^	5.85 ± 0.07 ^d^
Total	224.03 ± 2.17 ^d^	139.90 ± 0.36 ^e^	114.22 ± 1.02 ^f^	363.727 ± 4.30 ^c^	568.65 ± 4.67 ^b^	726.79 ± 3.19 ^a^
TPC	490.08 ± 5.47 ^e^	558.97 ± 2.81 ^d^	298.96 ± 2.41 ^f^	1243.48 ± 9.19 ^c^	1886.23 ± 11.20 ^a^	1721.57 ± 6.75 ^b^

Values are expressed as the mean ± standard deviation (*n* = 3). ND denotes not detected; SPs refer to soluble polyphenols. Superscript lowercase letters within the same row indicate statistically significant differences according to the Tukey test (*p* < 0.05).

**Table 4:** Identification of the polyphenols from IBPs in the extract of HB during in vitro digestion.

Identification	Indigestion		Gastrointestinal Digestion		Colonic Fermentation	
	Raw HB (μg/g DW)	Roasted HB (μg/g DW)	Raw HB (μg/g DW)	Roasted HB (μg/g DW)	Raw HB (μg/g DW)	Roasted HB (μg/g DW)
Hydroxycinnamic acid						
Sinapic acid	ND	ND	ND	ND	21.95±0.80	ND
*o*-Coumaric acid	ND	ND	ND	ND	ND	0.61 ± 0.05
*trans*-Cinnamic acid	16.11 ± 0.38 ^b^	1.18 ± 0.06 ^d^	2.87 ± 0.11 ^c^	ND	39.97 ± 0.78 ^a^	ND
Total	16.11 ± 0.38 ^b^	1.18 ± 0.06 ^d^	2.87 ± 0.11 ^c^	ND	24.81 ± 0.71 ^a^	0.61 ± 0.05 ^e^
Flavonoids						
Myricetin	ND	6.29 ± 0.44	ND	ND	39.97 ± 0.78	ND
Quercetin	ND	3.71 ± 0.35	ND	ND	65.56 ± 0.68	ND
Kaempferol	3.62 ± 0.27 ^d^	5.71 ± 0.24 ^c^	6.44 ± 0.27 ^b^	4.20 ± 0.11 ^d^	14.53 ± 0.34 ^a^	4.04 ± 0.23 ^d^
Apigenin	2.18 ± 0.15 ^c^	3.28 ± 0.11 ^b^	1.98 ± 0.19 ^c^	0.63 ± 0.01 ^d^	8.16 ± 0.17 ^a^	3.02 ± 0.18 ^b^
Total	5.80 ± 0.30 ^e^	18.99 ± 0.58 ^b^	8.43 ± 0.34 ^c^	4.83 ± 0.12 ^f^	128.22 ± 0.37 ^a^	7.06 ± 0.32 ^d^
TPC	38.02 ± 0.66 ^b^	21.36 ± 0.61 ^c^	8.43 ± 0.34 ^d^	4.83 ± 0.12 ^e^	177.86 ± 1.72 ^a^	8.29 ± 0.42 ^d^

Values are expressed as the mean ± standard deviation (*n* = 3). ND denotes not detected; SPs refer to insoluble bound polyphenols. Superscript lowercase letters within the same row indicate statistically significant differences according to the Tukey test (*p* < 0.05).

**Table 5:** In vitro digestion effects on α-amylase, α-glucosidase, and lipase inhibitory activities of Polyphenol Extracts from HB.

	IC50 (Mean ± SD in μg FAE/mL)						
	Indigestion		Gastrointestinal Digestion		Colonic Fermentation		Acarbose/Orlistat
	Raw HB	Roasted HB	Raw HB	Roasted HB	Raw HB	Roasted HB	
α-Amylase	107.24 ± 0.51 ^c^	82.13 ± 0.61 ^e^	89.31 ± 0.78 ^c^	71.31 ± 1.35 ^f^	120.65 ± 2.35 ^b^	87.51 ± 1.26 ^d^	133.25 ± 4.47 ^a^
α-GLU	98.17 ± 0.24 ^c^	81.35 ± 0.49 ^e^	82.20 ± 0.87 ^d^	60.44 ± 0.14 ^f^	108.94 ± 1.08 ^b^	95.31 ± 0.37 ^d^	133.25 ± 4.47 ^a^
Lipase	105.24 ± 2.49 ^b^	96.40 ± 1.19 ^c^	68.16 ± 1.87 ^c^	52.94 ± 2.51 ^d^	133.22 ± 0.46 ^a^	102.28 ± 2.20 ^b^	138.38 ± 2.39 ^a^

Superscript lowercase letters within the same row denote significant differences determined by Tukey’s test (*p* < 0.05).

## Data Availability

The original contributions presented in the study are included in the article. Further inquiries can be directed to the corresponding authors.
